# Supplementary material for: Identification of photoperiod-induced specific miRNAs in the adrenal glands of Sunite sheep (Ovis aries)
Source: Front Vet Sci. 2022 Jul 22;9:888207. doi: 10.3389/fvets.2022.888207 (PMC9354845; doi:10.3389/fvets.2022.888207)
Supplement: Supplementary file 1 [file Data_Sheet_1.ZIP › Supplementary file/miRDeep2 file.zip!/miRDeep2 file/Know_Structure/SPLP42A3/oar-let-7a.pdf]

miRBase precursor : oar-let-7a
Total read count : 271721
oar-let-7a read count : 270548
remaining reads : 1173

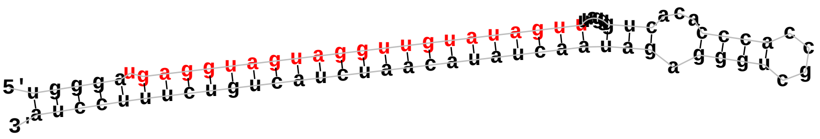

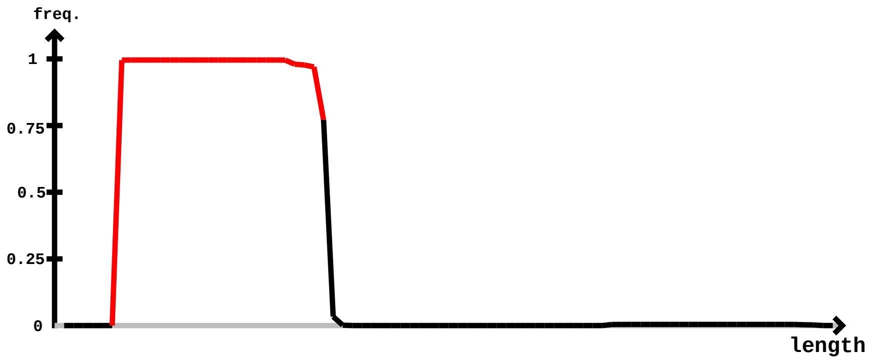


```
      oar-let-7a
5'- ugggaugagguaguagguuguauaguuuuagggucacacccaccgcugggagauaacuauacaaucuacugucuuuccua   -3'    exp
    (((((.(((((((((((((((((((((.....(((...((((....)))).)))))))))))))))))))))))))))))    reads    mm        sample
    ....augagguaguagguuguauagu......................................................    3        0         seq
    ....Uugagguaguagguuguauagu......................................................    2        1         seq
    ....augagguaguagguuguauaguu.....................................................    10       0         seq
    ....Uugagguaguagguuguauaguu.....................................................    7        1         seq
    .....ugagguaguagguugua..........................................................    54       0         seq
    .....ugagguaguaAguugua..........................................................    1        1         seq
    .....ugagguagGagguugua..........................................................    16       1         seq
    .....ugagguagGagguuguau.........................................................    337      1         seq
    .....ugagguaguagguuguau.........................................................    3808     0         seq
    .....ugagguaguaAguuguau.........................................................    12       1         seq
    .....ugagguaguagguuUuau.........................................................    16       1         seq
    .....ugagguUguagguuguau.........................................................    4        1         seq
    .....ugaAguaguagguuguau.........................................................    30       1         seq
    .....ugagguagGagguuguaua........................................................    109      1         seq
    .....ugagguaguagguuguaua........................................................    651      0         seq
    .....ugagguaguagguuguauag.......................................................    1620     0         seq
    .....ugagguagGagguuguauag.......................................................    386      1         seq
    .....ugagguaguagguuguauagu......................................................    47796    0         seq
    .....ugagguagGagguuguauagu......................................................    6036     1         seq
    .....ugagguaguagguuguauaguu.....................................................    184384   0         seq
    .....ugagguagGagguuguauaguu.....................................................    16038    1         seq
    .....ugagguaguagguuguauaguuu....................................................    8712     0         seq
    .....ugagguaguagguuguauaguuuA...................................................    92       1         seq
    .....ugagguaguagguuguauaguuuu...................................................    280      0         seq
    .....ugagguaguagguuguauaguuuua..................................................    3        0         seq
    ......gagguaguagguuguaua........................................................    1        0         seq
    ......gagguaguagguuguauagu......................................................    28       0         seq
    ......gagguagGagguuguauagu......................................................    1        1         seq
    ......gagguaguagguuguauaguu.....................................................    96       0         seq
    ......gagguagGagguuguauaguu.....................................................    5        1         seq
    ......gagguaguagguuguauaguuu....................................................    5        0         seq
    .......agguaguagguuguauagu......................................................    1        0         seq
    ........gguaguagguuguauaguu.....................................................    1        0         seq
    ..........uaguagguuguauaguuuu...................................................    2        0         seq
    ............guagguuguauGguuuu...................................................    1        1         seq
```

oar-let-7a

```
ugggaugagguaguagguuguauaguuuuagggucacacccaccgcugggagauaacuauacaaucuacugucuuuccua
...........................uuagggucacacccaccgcuggg..............................    1      0      seq
...........................uuagggucacacccaccgcuggga.............................    7      0      seq
...........................uuagggucacacccaccgcugggagau..........................    4      0      seq
....................................................auaacuauacaaucuacugucuu.....    1      0      seq
.....................................................uaacuauacaaucuacugucu......    1      0      seq
.....................................................uaacuauacaaucuacugucuuu....    6      0      seq
......................................................aacuauacaaucuacugucuuu....    2      0      seq
.......................................................acuauacaaucuacugucuuu....    1      0      seq
........................................................cuauacaaucuacugucuu.....    7      0      seq
........................................................cuauacaauUuacugucuuu....    1      1      seq
........................................................cuauacaaucuacugucuuu....    304    0      seq
........................................................cuauacaaucuacuguUuuuc...    3      1      seq
........................................................cuauacaaucuacugucuuuc...    172    0      seq
........................................................cuauacaaucuacugucuuucc..    595    0      seq
........................................................cuauacaaucuacugucuuuccu.    30     0      seq
.........................................................uauacaaucuacugucuuu....    3      0      seq
.........................................................uauacaaucuacugucuuuc...    2      0      seq
.........................................................uauacaaucuacugucuuucc..    2      0      seq
.........................................................uauacaaucuacugucuuuccu.    23     0      seq
..........................................................auacaaucuacugucuuucc..    1      0      seq
..........................................................auacaaucuacugucuuuccu.    6      0      seq
...........................................................uacaaucuacugucuuuccu.    1      0      seq
```
